# Three-dimensional pharyngeal airway space changes after bimaxillary advancement

**DOI:** 10.1590/2177-6709.26.5.e2119364.oar

**Published:** 2021-10-15

**Authors:** Thaís Lima ROCHA, Ludmila LIMA, Arnaldo PINZAN, Eduardo SANT’ANA, Renato Luiz Maia NOGUEIRA, Caroline Nemetz BRONFMAN, Guilherme JANSON

**Affiliations:** 1 Universidade de São Paulo, Faculdade de Odontologia de Bauru, Departamento de Ortodontia (Bauru/SP, Brazil).; 2 Universidade de São Paulo, Faculdade de Odontologia de Bauru, Departamento de Estomatologia (Bauru/SP, Brazil).; 3 Universidade Federal do Ceará, Faculdade de Odontologia, Departamento de Cirurgia Oral (Fortaleza/CE, Brazil).

**Keywords:** Bimaxillary advancement, Orthognathic surgery, Upper airway space, Cone-beam computed tomography, Obstructive sleep apnea

## Abstract

**Introduction:**

The probability of improvement in the upper airway space (UAS) with orthognathic surgery should be considered during the surgical-orthodontic treatment decision, providing not only an esthetic, but also a functional benefit for the patient.

**Objective:**

The purpose of this study was to evaluate the 3D changes in the upper airway space after maxillomandibular advancement surgery (MMA).

**Methods:**

A retrospective analysis of 56 patients, 21 male and 35 female, with a mean age of 35.8 ± 10.7 years, who underwent MMA was performed. Pre- and postoperative cone-beam computed tomography scans (CBCT) were obtained for each patient, and the changes in the UAS were compared using Dolphin Imaging 11.7 software. Two parameters of the pharyngeal airway space (PAS) were measured: airway volume (AV) and minimum axial area (MAA). Paired *t*-test was used to compare the data between T_0_ and T_1_, at 5% significance level.

**Results:**

There was a statistically significant increase in the UAS. Bimaxillary advancement surgery increased the AV and the MAA, on average, by 73.6 ± 74.75% and 113.5 ± 123.87%, respectively.

**Conclusion:**

MMA surgery tends to cause significant increase in the UAS; however, this increase is largely variable.

## RESUMO

**Introdução:**

A probabilidade de melhoria do espaço aéreo superior (EAS) com cirurgia ortognática deve ser considerada durante a decisão do tratamento ortodôntico-cirúrgico, proporcionando não somente um benefício estético, mas também funcional, para o paciente.

**Objetivo:**

O objetivo do presente estudo foi avaliar as alterações 3D no espaço das vias aéreas superiores após a cirurgia de avanço maxilomandibular (AMM).

**Métodos:**

Foi realizada uma análise retrospectiva de 56 pacientes, 21 homens e 35 mulheres, com média de idade de 35,8 ± 10,7 anos, submetidos a AMM. Foram obtidas tomografias computadorizadas de feixe cônico (TCFC) pré- e pós-operatórias para cada paciente, e as alterações no EAS foram comparadas usando o *software* Dolphin Imaging v. 11.7. Foram medidos dois parâmetros do espaço aéreo faríngeo (EAF): volume das vias aéreas (VVA) e área axial mínima (AAM). Foi utilizado o teste *t* pareado para comparar os dados entre T_0_ e T_1_, com nível de significância de 5%.

**Resultados:**

Houve um aumento estatisticamente significativo no EAS. A cirurgia de avanço bimaxilar aumentou o volume das vias aéreas (VVA) e a área axial mínima (AAM) em média 73,6 ± 74,75% e 113,5 ± 123,87%, respectivamente.

**Conclusão:**

A cirurgia de AMM tende a causar o aumento significativo do EAS; no entanto, esse aumento é altamente variável.

## INTRODUCTION

Harmonious facial esthetics and great functional occlusion have been recognized as the two most important goals of orthodontic treatment. For the correct indication of treatment, an accurate malocclusion and skeletal discrepancy diagnosis is needed. This care leads to adequate planning and multidisciplinary treatment with the objective of an esthetic and functional correction.^1^

Dissatisfaction with facial esthetics is considered the most common motivating factor in the search for orthognathic surgery, since this is the procedure indicated in cases of severe dental and skeletal discrepancies in adult patients.^2^

Airways effects caused by skeletal movements of the basal bones after orthognathic surgery are essential because they produce a change in the position of the hyoid bone and tongue.^3^

Upper Airway Space (UAS) is formed by soft tissue structures: tonsils, soft palate, uvula, tongue and lateral pharyngeal wall. The mandible and the hyoid bone are the main craniofacial bone structures that determine the airway size. Thus, the UAS anatomical conformation allows factors such as obesity, muscle hypotonicity and mandibular deficiency to favor the obstruction, generating Obstructive Sleep Apnea (OSA), which has been the subject of numerous studies.^4^ ^-^ ^7^

OSA is characterized by recurrent episodes of partial or complete upper airway (UA) obstruction during sleep. The airflow is reduced in hypopnea or completely interrupted in apnea. These respiratory events are normally interrupted by micro-arousals. According to the American Academy of Sleep Medicine (AASM) criteria, to diagnose OSA it is necessary that the patient presents the following symptoms: excessive daytime sleepiness not explained by other factors, choking during sleep, recurrent awakenings, non-repairing sleep, daytime fatigue or difficulty in concentrating, and polysomnographic monitoring overnight showing five or more obstructive respiratory events per hour of sleep.^8^

Several factors can aggravate or predispose to sleep disorders. Changes in the upper airway space caused by orthognathic surgery have been a concern, because the quality of sleep can be increased or aggravated by these changes. The main concern involving these dimensional changes caused by orthognathic surgery is the sleep quality.^3^ ^,^ ^9^ ^-^ ^11^

Thus, the orthodontist should be aware of changes that may occur in the upper airway before proposing orthognathic surgery for patients. It is important to assess whether the patient with mandibular retrusion has associated symptoms of obstructive sleep apnea, such as obesity, excessive daytime sleepiness and snoring. The reason for this is that the possibility of improvement or not with orthognathic surgery should be considered during the decision for surgical orthodontic treatment, providing not only esthetic but also functional benefits for the patient.^3^ ^,^ ^9^ ^,^ ^12^ ^,^ ^13^

Although there is clear evidence that bimaxillary advancement surgery can effectively increase the upper airway,^14^ ^,^ ^15^ most studies have a limited number of patients.^16^ ^-^ ^20^ Besides, they have not individually quantified the amount and percentages of upper air volume and minimum axial area increase. Therefore, the purpose of this study is to evaluate, in 3D images, the changes in the pharyngeal airway space (PAS) in skeletal Class I or Class II malocclusion patients, submitted to bimaxillary advancement surgery using bilateral sagittal split osteotomy for mandibular advancement, associated with maxillary advancement with Le Fort I maxillary osteotomy.

## MATERIAL AND METHODS

This study was approved by the Ethics in Research Committee at *Faculdade de Odontologia de Bauru* (FOB-USP, Brazil), under protocol number 48092215.0.0000.5417.

Using an alpha error of 5% and a beta error of 20%, considering a standard deviation of 37%, to detect a minimum difference of 10% for the volumetric pharyngeal space variable, the results indicated that a minimum of 55 patients was necessary.^13^

A retrospective analysis of 56 patients (35 female, 21 male), with a mean age of 35.8 ± 10.7 years, who underwent bimaxillary advancement orthognathic surgery due to functional and esthetic complaints, was performed. The sample was selected to be as homogeneous as possible. Inclusion criteria consisted of adult patients of both sexes diagnosed primarily with skeletal Class II and some with skeletal Class I malocclusion, submitted to bimaxillary advancement surgery. These patients did not have a documented OSA diagnosis and had no respiratory indications for surgery. Patients with severe facial asymmetry, transverse discrepancy of the maxilla, presence of syndromes, temporomandibular joint disorder or degeneration, and incomplete records were excluded. Sample characteristics regarding sex and age are presented in Table 1.

**Table 1:** Sample distribution by sex and age.

	Skeletal Class I			Skeletal Class II		Total sample	
	n	%	n	%	n	%
Sex	18	32.1	38	67.9	56	100
Female	5	8.9	30	53.6	35	62.5
Male	13	23.2	8	14.3	21	37.5
*p* < 0.001^†^*						
	Mean	SD	Mean	SD	Mean	SD
Age	38.43	10.40	38.72	10.76	38.63	10.55
*p* = 0.924^‡^						

^†^Chi-square test; ^‡^ *t*-test; *Statistically significant at *p*< 0.05.

All procedures were performed by the same surgeon, who performed the maxillary advancement using a Le Fort I maxillary osteotomy, and the mandibular advancement using bilateral sagittal split osteotomy technique, with rigid fixation of the bone segments. The amount of advancement was planned using Arnett’s soft tissue cephalometric analysis.^21^ The patients had a mean maxillary advancement of 3.27 ± 3.24 mm, and a mean mandibular advancement of 9.41 ± 4.26 mm. There was also a mean maxillary intrusion of -1.3 ± 4.3 mm and a mean mandibular downward movement of 0.53 ± 5.19 mm. Horizontal displacements were measured from A and B points to a line parallel to the true vertical, through Sella; and vertical displacements were measured from A and B points to a perpendicular line to the true vertical, through Nasion. All patients received routine postoperative orthodontic treatment.

Every patient underwent a preoperative CBCT at the end of the presurgical orthodontic treatment, and a postoperative CBCT at the follow-up visit, after a mean of 8.43 months after surgery. In each case, CBCT was performed with the i-CAT (Imaging Science, Hatfield, PA, USA). The scanning speed was 40 s, and high-resolution images were obtained. The radiologic parameters were 120 KpV, 36.90 µSv, and a voxel size of 0.4 mm. During the CBCT, each patient was carefully instructed to be seated, with the Frankfurt horizontal plane parallel to the floor, the head in natural position, to breathe quietly and not to swallow during the scan. The images were then stored as Digital Imaging and Communications in Medicine (DICOM) data files.

Each CBCT scan was processed using Dolphin Imaging software version 11.7 (Dolphin Imaging and Management Solutions, Patterson Dental Supply, Inc., Chatsworth, CA). The area of interest for the upper airway evaluation was defined as the velopharynx, oropharynx and hypopharynx. The limits of the UAS used in this study were two lines: the upper line, passing through the post-palatal area,; and the lower line, passing through the post-glossal area. The landmarks used were Posterior Nasal Spine (PNS) - point at posterior edge of the nasal spine; CV_2_- point of the top of the body of the second cervical vertebra; CV_3_- lower posterior point of the body of the third cervical vertebra; Hyoid bone (H) - posterior superior point of the hyoid bone. The area of interest was defined by a clipping box and seeds in the airway space.

**Figure 1:**
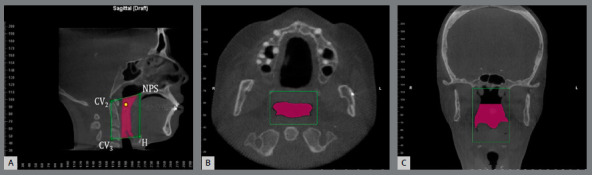
Upper airway volumetric measurement. A) Limits of retropalatal and retroglossal areas, in sagittal view. B, C) The corresponding limits in the axial and coronal views, respectively. Pink areas denote defined airway portion of interest.

Once the portion of the airway of interest was defined, the Dolphin 3D airway analysis tool was used to define and measure two parameters of the pharyngeal airway space (PAS): airway volume (AV) and minimum axial area (MAA). Each patient’s UAS measurements, before and after surgery, were then compared (Fig 1).

### ERROR STUDY

Twenty CBCT were randomly selected and remeasured by the same examiner after a 15-day interval. The random errors were calculated according to Dahlberg’s formula, S^2^ = Σd^2^/2n, where S^2^ is the error variance and *d* is the difference between two determinations of the same variable; and the systematic errors were estimated with dependent *t*-tests, at *p*< 0.05.^22^ ^,^ ^23^

### STATISTICAL ANALYSES

Kolmogorov-Smirnov tests were used to test the normal distribution of the variables.

Pre- and postoperative data comparisons of airway volume and minimum cross-sectional area of the upper airways were performed with paired *t*-tests. The influence of maxillary and mandibular advancement in the changes of airway volume and minimum axial area were evaluated with multiple linear regression analyses. Airway changes comparisons considering the skeletal sagittal relationship (Class I *vs.* Class II) and sex (Female *vs.* Male) as subgroups were performed with Mann-Whitney U tests.

The statistical analyses were performed with Statistica software (Statistica 7, StatSoft Inc., Tulsa, OK). Results were considered significant at *p*< 0.05.

## RESULTS

The random errors were within acceptable limits^24^ ^,^ ^25^ (AV = 686.48mm^3^; MAA = 0.21mm^2^), and there was no significant systematic error for both variables (*p-*values were 0.155 and 0.468 for AV and MAA, respectively). 

There were significant increases in volume and minimum axial area in the airways after surgery (Table 2). The mean percentage of changes in the AV and MAA were 73.6% (SD = 74.75; Min. = 10.6; Max. = 447.0) and 113.5% (SD = 123.87; Min. = -42.7; Max. = 555.3), respectively.

**Table 2:** Intragroup airway volume and minimum axial area changes with the surgical procedure (paired *t*-tests, n = 56).

	Preoperative (T_0_)		Postoperative (T_1_)		Mean difference (T_1_ - T_0_)	*p*	95% CI
	Mean	SD	Mean	SD			
AV (mm^3^)	13392.07	6235.74	21133.29	7922.92	7741.22	0.000*	6024.82 - 9457.63
MAA (mm^2^)	142.33	86.35	251.30	126.25	108.97	0.000*	79.67 - 138.27

* Statistically significant at *p*< 0.05. AV: airway volume. MAA: minimum axial area.

The amount of maxillary and mandibular advancement did not show significant influence on the airway volume and minimum axial area (Table 3).

**Table 3:** Multiple linear regression analyses considering maxillary and mandibular advancements as predictors, and airway volume (AV) and minimum axial area (MAA) changes as outcome variables.

Variables	AV (mm^3^) change				MAA (mm^2^) change			
	B	P	95% CI		B	P	95% CI	
			Lower limit	Upper limit			Lower limit	Upper limit
Constant	6866.72	0.003	2428.28	11305.16	135.05	0.001	59.53	210.58
Maxillary advancement	66.84	0.825	-537.96	671.66	-0.03	0.995	-10.32	10.26
Mandibular advancement	68.71	0.773	-407.59	545.01	-2.72	0.503	-10.82	5.38

AV (mm^3^) change, r^2^ = 0.004, P = 0.890; MAA (mm^2^) change, r^2^ = 0.011, *p*= 0.754.

Similar airway volume and minimum axial area changes were observed between skeletal Class I and Class II, and between female and male patients (Tables 4 and 5).

**Table 4:** Airway volume (AV) and minimum axial area (MAA) changes comparison regarding skeletal sagittal relationship (Mann-Whitney U test).

	Skeletal Class I (n=18)		Skeletal Class II (n=38)		Mean difference	P	95% CI
	Mean	SD	Mean	SD			
AV (mm^3^) change	8050.70	4189.07	7594.62	7275.35	456.07	0.362	-3252.43 - 4164.59
MAA (mm^2^) change	121.92	90.81	102.82	117.82	19.09	0.425	-44.03 - 82.21

**Table 5:** Airway volume (AV) and minimum axial area (MAA) changes comparison regarding sex (Mann-Whitney U test).

	Female (n=35)		Male (n=21)		Mean difference	P	95% CI
	Mean	SD	Mean	SD			
AV (mm^3^) change	8261.78	7248.16	6873.62	4731.74	1388.15	0.630	-2171.30 - 4947.61
MAA (mm^2^) change	114.02	121.83	100.52	86.97	13.50	0.986	-47.49 - 74.49

Table 6 displays the number of patients according to the percentage of changes in the airway volume and minimum axial area.

**Table 6:** Number of patients according to the percentage of changes in the minimum axial area and airway volume.

MAA and AV Range of % of change between T_0_ and T_1_ (difference value/initial value x 100)	n (AV)	% AV in relation to the total sample (n = 56)	n (MAA)	% MAA in relation to the total sample (n = 56)
-40 < X ≤ 0	1	1.8%	4	7.1%
0 < X ≤ 25	11	19.6%	6	10.7%
25 < X ≤ 50	16	28.6%	5	8.9%
50 < X ≤ 75	9	16.1%	12	21.4%
75 < X ≤ 100	7	12.5%	9	16.1%
100 < X ≤ 200	9	16.1%	10	17.9%
X > 200	3	5.3%	10	17.9%
TOTAL	56	100%	56	100%

## DISCUSSION

The present study only verified CT scans taken at the postoperative stage at a mean of 8.43 months. The reduction in airway space in the immediate postoperative period may occur as a consequence of edema, masking the actual gain in airway space. Edema is an important factor in the evaluation of airway space, particularly in the immediate postoperative period of maxillomandibular advancement surgery.^9^ ^,^ ^26^ ^,^ ^27^ It was observed that the difference in time of follow up between the studies was quite variable, from 6 weeks to 12 years, constituting a bias in possible comparisons between studies. This type of assessment is not performed due to the ethical issues involved in exposing patients to unnecessary radiation.^14^ The most common period of follow-up was 6 months.^3^ ^,^ ^9^

Patients in the present sample had a mean mandibular advancement of 9.41 ± 4.26 mm. Bimaxillary advancement surgery performed an important role in the OSA correction when medical treatment is not tolerated and in patients who wish a definitive correction, whereas this surgery with an advancement greater than 10 mm is considered effective to improve OSAS.^26^ Based on the common perception and the literature, older adult patients usually require advancement of 10 mm.^16^ ^,^ ^28^ ^-^ ^30^

Even with the increasing number of 3D studies evaluating the airways, the great variability in the choice of airway delimitation landmarks makes it difficult to compare them. Posterior nasal spine (PNS) was used as the anterior limit of the airway space for volumetric measurements, as performed in other studies.^31^ ^-^ ^33^ Hyoid bone and PNS were used because they are hard tissues, which consist of more precise and consistent form of identification, compared to soft tissue palate and epiglottis, which could vary after surgery.^25^ ^,^ ^30^ ^,^ ^34^ The different measurements adopted by the authors to evaluate the oropharyngeal airway changes make it impossible to compare all studies among themselves, regardless of the type of surgery adopted.^3^ ^,^ ^14^ PNS was used as the airway limit for volumetric measurements, as in most studies.^25^ ^,^ ^32^ ^,^ ^33^ ^,^ ^35^ Small variations in the anatomical limits and calibration and training of examiners did not seem to have great influence on the results.^14^ ^,^ ^26^ The present study evaluated only the changes in the oropharyngeal region, due to the difficulty of evaluating the nasopharyngeal region. In a study evaluating the reliability and accuracy of airway measurement in three dimensions of three different software, the authors observed a precision discrepancy in the volume quantification between the different evaluated software. According to them, the nasopharyngeal volume evaluation was more challenging and showed lower reliability, due to the presence of some anatomical structures (turbinate and the concha region) that create intricate anatomy.^35^ For the oropharyngeal evaluation, there was a smaller difference in the results found in different software.^9^

Dolphin 3D software was used because it showed high accuracy and reliability for the volumetric assessment of airspace in previous studies, and was therefore used in this study.^9^ ^,^ ^26^ ^,^ ^35^ ^,^ ^36^ This software provides greater accuracy because it is a tool for inclusion of reference points in the images, which allows quantification control of volume limits, with few errors (1%).^35^ ^,^ ^36^ Variations in the soft palate and tongue positions between pre- and post-surgical exams may significantly influence the outcome of this variable.^26^ Thus, patients who presented visible differences in the position of these structures in T_0_ and T_1_ periods were excluded from the sample.

The literature shows that there is no difference in the upper airway when comparing patients with Class I and Class II malocclusion, unlike the patient with Class III malocclusion.^37^ In the present study, there was significant increase in the airway volume and minimum axial area in almost all patients, regardless of sex and sagittal relationship (Tables 2, 3 and 4). These variables were analyzed to indirectly contribute to the surgical treatment of patients with OSA. Many surgical treatments used for patients with OSA, such as turbinectomies, uvulopalatopharyngoplasty, and reduction glossectomies, are associated with low success rates, between 17% and 40%, when performed alone, because they act only on the airway obstruction.^38^ ^-^ ^42^ Bimaxillary advancement has the benefit of optimizing airway gain, increasing success rate in OSA treatment, and correcting the patients’ dentofacial and esthetic deformities.^43^ ^,^ ^44^

Although a retrusive craniofacial profile is predictive of OSA, there is still controversy among authors.^45^ ^,^ ^46^ Comparisons performed at the preoperative stage between OSA patients and control patients without OSA showed significant less volume in the OSA group, as expected. Nevertheless, the control group without OSA had relatively (but not statistically significant) more bimaxillary retrusion, when compared with the OSA group, indicating that the craniofacial profile may not reliably predict the presence of OSA.^19^

In this research, bimaxillary advancement surgery provided significant volumetric increases in the upper airways, as well as in the minimal axial area, corroborating with the literature.^13^ ^,^ ^18^ After assessing the airway morphological changes, the bimaxillary advancement leads to airway increase in all dimensions, anteroposterior or latero-medial.^25^ ^,^ ^26^ ^,^ ^47^ ^,^ ^48^ Another study observed statistically significant increases in all airway dimensions in the analysis of minimal axial area and volume,^9^ and in the oropharyngeal airway at the soft palate level.^3^ Some studies have evaluated the effects of single-jaw orthognathic procedures on the upper airways, and have also found significant increases in upper airway volume.^17^ ^,^ ^49^ ^,^ ^50^

There was no volumetric gain in the oropharyngeal region in only one patient of the sample (Table 5). This can occur because bimaxillary advancement causes an increase in airway width, decreasing its constriction and air passage resistance, and may lead to a decrease in height in this area.^47^ In the current study, nine patients had MAA values ​​below 67mm², and presented a postoperative mean gain of 143.26% (Table 5). There is a statistically significant relationship between the narrower cross section of the upper airway and the OSA probability. Small airway area of about 40 to 67mm² is associated with OSA,^30^ so the patients in this sample left the range of predisposition to OSA.

In this research, the minimum axial area and volume presented considerable gains. One study concluded that the airway resistance decrease after this type of surgery was secondary to a shorter and wider area.^16^ Poiseuille’s law demonstrates that as the radius of a tube (or an airway) increases and height decreases, there is a resulting significant decrease in airway resistance.^16^ ^,^ ^51^ Based on this evidence, it could be thought that increasing the surface area due to increases in anteroposterior and transverse dimensions could lead to a decrease in airway resistance. Despite this outcome, four patients presented a decrease in MAA (Table 6); yet, still maintaining normal values. Individual anatomical changes and soft tissue adaptations (hyoid bone position, pharyngeal airway space narrowing and tongue position) may justify this decrease.^30^ ^,^ ^52^ ^,^ ^53^

Due to a representative number of patients, it was possible to ascertain that bimaxillary advancement actually produces significant increase in the UAS regardless of sex and skeletal sagittal relationship, and to individually quantify the amount and percentages of AV and MAA increases. 

Adequate surgical planning considers the airways, masticatory function, occlusion and esthetics. Proper management of all four variables leads to success.^25^ ^,^ ^54^ CBCT generalized use and the recent development of automated airway analysis systems that have been validated allow a more refined surgical planning by the surgeon, since exact locations and extent of obstruction can best be visualized. Thus, the surgery can be individualized for each patient.^30^ ^,^ ^55^

In addition, OSA has a multifactorial etiology; thus, static airway morphology is not the only factor that contributes to its manifestation. The airway is a dynamic biological structure subjected to various hormonal, neuromuscular and biomechanical influences, which are also factors that may play a role in the OSA pathophysiology.^56^ ^,^ ^57^ However, bimaxillary advancement surgery provides anatomical and/or structural improvement of the pharyngeal airway in patients with OSA, but other contributing factors should also be considered to influence the OSA presence and severity.^41^ It is necessary to consider the possibility of gain in the upper airway volume and MAA, in the treatment of patients with different malocclusions, especially those with mandibular retrusion likely to have a minor oropharyngeal region. For this reason, a detailed analysis of the volume and airways shape, with cephalometric evaluations, may prove to be a valuable diagnostic addition to Orthodontics. As a result, balance between function restoration and esthetic optimization is extremely important in the treatment of these types of patients.

## LIMITATIONS

The main limitation of this retrospective study was the great variability in the amount of maxillary and mandibular vertical and horizontal surgical displacements, due to including skeletal Class I and Class II malocclusions patients.

## CONCLUSIONS

Bimaxillary advancement surgery to correct skeletal Class I and Class II malocclusions had a tendency to produce significant increase in the UAS (AV and MAA).

However, the amount of increase in the UAS, with bimaxillary advancement surgery in Class I and Class II malocclusions patients, widely varied.
